# Pyridostigmine reduces mortality of patients with severe SARS-CoV-2 infection: A phase 2/3 randomized controlled trial

**DOI:** 10.1186/s10020-022-00553-x

**Published:** 2022-11-08

**Authors:** Sergio Fragoso-Saavedra, Isaac Núñez, Belem M. Audelo-Cruz, Sarahi Arias-Martínez, Daniel Manzur-Sandoval, Alejandro Quintero-Villegas, H. Benjamín García-González, Sergio L. Carbajal-Morelos, Sergio PoncedeLeón-Rosales, José Gotés-Palazuelos, José A. Maza-Larrea, J. Javier Rosales-de la Rosa, Dafne Diaz-Rivera, Edgar Luna-García, Elvira Piten-Isidro, Perla M. Del Río-Estrada, Mario Fragoso-Saavedra, Yanink Caro-Vega, Isabella Batina, León Islas-Weinstein, David A. Iruegas-Nunez, Juan J. Calva, Pablo F. Belaunzarán-Zamudio, Juan Sierra-Madero, José C. Crispín, Sergio Iván Valdés-Ferrer

**Affiliations:** 1 Plan de Estudios Combinados en Medicina (MD/PhD Program), Facultad de Medicina, Universidad Nacional Autónoma de México, Mexico City, Mexico; 2 Programa de Residencia en Medicina Interna, Instituto Nacional de Ciencias Médicas y Nutrición Salvador Zubirán, Mexico City, Mexico; 3 Departamento de Educación Médica, Instituto Nacional de Ciencias Médicas y Nutrición Salvador Zubirán (INCMNSZ), Mexico City, Mexico; 4 Escuela Superior de Medicina, Instituto Politécnico Nacional, Mexico City, BM Mexico; 5 Instituto Nacional de Cardiología Ignacio Chávez (INCICh), Mexico City, Mexico; 6 Facultad de Medicina, Universidad Autónoma de Coahuila, Torreón, Mexico; 7 Centro de Investigación y de Estudios Avanzados del Instituto Politécnico Nacional, Departamento de Infectómica y Patogénesis Molecular, Mexico City, Mexico; 8 Departamento de Neurología y Psiquiatría, Instituto Nacional de Ciencias Médicas y Nutrición Salvador Zubirán, Mexico City, Mexico; 9 División de Medicina, Instituto Nacional de Ciencias Médicas y Nutrición Salvador Zubirán, Mexico City, Mexico; 10 Departamento de Inmunología y Reumatología, Instituto Nacional de Ciencias Médicas y Nutrición Salvador Zubirán, Mexico City, Mexico; 11 Departamento de Investigación en Enfermedades Infecciosas, Instituto Nacional de Enfermedades Respiratorias, Mexico City, Mexico; 12 Departamento de Infectología, Instituto Nacional de Ciencias Médicas y Nutrición Salvador Zubirán, Mexico City, Mexico; 13 Departamento de Patología Experimental, Instituto Nacional de Ciencias Médicas y Nutrición Salvador Zubirán, Mexico City, Mexico; 14 Escuela de Medicina y Ciencias de la Salud, Tecnologico de Monterrey, Monterrey, Mexico

**Keywords:** COVID-19, SARS-CoV-2, Mortality, Invasive mechanical ventilation, Immunomodulation, Pyridostigmine, ACh, Inflammatory reflex, Placebo-controlled trial

## Abstract

**Background:**

Respiratory failure in severe coronavirus disease 2019 (COVID-19) is associated with a severe inflammatory response. Acetylcholine (ACh) reduces systemic inflammation in experimental bacterial and viral infections. Pyridostigmine increases the half-life of endogenous ACh, potentially reducing systemic inflammation. We aimed to determine if pyridostigmine decreases a composite outcome of invasive mechanical ventilation (IMV) and death in adult patients with severe COVID-19.

**Methods:**

We performed a double-blinded, placebo-controlled, phase 2/3 randomized controlled trial of oral pyridostigmine (60 mg/day) or placebo as add-on therapy in adult patients admitted due to confirmed severe COVID-19 not requiring IMV at enrollment. The primary outcome was a composite of IMV or death by day 28. Secondary outcomes included reduction of inflammatory markers and circulating cytokines, and 90-day mortality. Adverse events (AEs) related to study treatment were documented and described.

**Results:**

We recruited 188 participants (94 per group); 112 (59.6%) were men; the median (IQR) age was 52 (44–64) years. The study was terminated early due to a significant reduction in the primary outcome in the treatment arm and increased difficulty with recruitment. The primary outcome occurred in 22 (23.4%) participants in the placebo group vs. 11 (11.7%) in the pyridostigmine group (hazard ratio, 0.47, 95% confidence interval 0.24–0.9; *P* = 0.03). This effect was driven by a reduction in mortality (19 vs. 8 deaths, respectively).

**Conclusion:**

Our data indicate that adding pyridostigmine to standard care reduces mortality among patients hospitalized for severe COVID-19.

## Background

Coronavirus disease 2019 (COVID-19), caused by severe acute respiratory syndrome coronavirus 2 (SARS-CoV-2), is a disease that ranges from asymptomatic to lethal (Zhu et al. 2020). Patients with COVID-19 may develop severe systemic inflammatory response and acute respiratory distress syndrome (ARDS), which may progress to multiple organ failure and death (Xu et al. 2020; Huang et al. 2020; Ruan et al. 2020; Mehta et al. 2020). Although determinants of COVID-19 severity are incompletely understood, the inflammatory response is crucial in the pathogenesis of the severe disease, making it a potential therapeutic target. Therefore, pharmacological strategies to reduce inflammation have been evaluated, showing diverse clinical success. For instance, dexamethasone reduces mortality among patients requiring supplementary oxygen or invasive mechanical ventilation (IMV) (Horby et al. 2021a, b). Baricitinib is currently the only immunomodulator approved for the treatment of severe COVID-19(Kalil et al. 2021). Other anti-inflammatory strategies, including tocilizumab, and tofacitinib, may reduce mortality of severe COVID-19, but high cost and technical needs limit their widespread use (RECOVERY Collaborative Group 2021; Guimarães et al. 2021; Beigel et al. 2020). Despite therapeutic advances, severe COVID-19 still has an elevated mortality rate, especially in countries with resource-limited settings and healthcare strain, highlighting the need for safe, easy-to-use, and inexpensive treatments (Cifuentes-Faura 2021).

The central nervous system regulates the inflammatory response through the so-called *inflammatory reflex*. In this pathway, acetylcholine (ACh) stimulates α-7 nicotinic ACh receptors (α7-nACh-R) on the surface of macrophages and other immune cells and its signaling decreases the production of inflammatory mediators (Borovikova et al. 2000; Pavlov and Tracey 2017; Lehner et al. 2019). Choline-acetyltransferase (ChAT)-expressing T-cells modulate inflammation via in situ release of ACh, and this effect is critical in reducing viremia in experimental lymphocytic choriomeningitis virus infection (Rosas-Ballina et al. 2011; Cox et al. 2019). Pyridostigmine is an acetylcholinesterase inhibitor (i-ACh-e) that increases ACh half-life by inhibiting its peripheral degradation. For this reason, pyridostigmine is used for the treatment of myasthenia gravis and as pre-exposure prophylaxis for nerve gas poisoning (Gilhus and Verschuuren 2015; Keeler et al. 1991). In human immunodeficiency virus (HIV)-1 infection, pyridostigmine modulates T-cell activation and reduces circulating inflammatory markers (Valdés-Ferrer et al. 2009, 2017; Robinson-Papp et al. 2019). In murine models of endotoxin-induced ARDS, an intact inflammatory reflex, and its induction through administration of α7-nACh-R agonists and pyridostigmine are crucial to reducing lung and systemic inflammation and decreasing mortality (Su et al. 2010; Pinheiro et al. 2021; Bricher Choque et al. 2021). Although pyridostigmine has not been evaluated as an immuno-modulator in acute severe human inflammatory conditions, the aforementioned clinical and experimental evidence suggests that it may effectively reduce systemic inflammation, which can be beneficial in patients with COVID-19.

In this study, we evaluated the efficacy of pyridostigmine as an adjunct therapy to reduce the incidence of IMV or death in hospitalized adults with severe COVID-19.

## Methods

### Study design and participants:

We performed a double-blinded, parallel group randomized, placebo-controlled, phase 2/3 trial at Instituto Nacional de Ciencias Médicas y Nutrición Salvador Zubirán and Instituto Nacional de Cardiología Ignacio Chávez, two COVID-19-designated hospitals in Mexico City, Mexico. Adult (≥ 18-year-old) hospitalized patients with positive nasopharyngeal SARS-CoV-2 real-time polymerase-chain-reaction (RT-PCR) test and imaging study compatible with pneumonia, and at least one risk factor for requiring IMV or dying, including hypoxemia, were invited to participate as previously described and published in the study protocol (Fragoso-Saavedra et al. 2020). The predominant SARS-CoV-2 variants during the study period in Mexico were B.1 and B.1.1 for the year 2020, and B.1.1.519 at the beginning of 2021 (Taboada et al. 2021). While concomitant corticosteroid use was part of the exclusion criteria, the results of the RECOVERY Collaborative trial made these standards of care; therefore, after recruiting the first 44 participants, the protocol was amended to include patients regardless of concomitant corticosteroid use (Horby et al. 2021a, b).

All participants provided written informed consent. The study protocol was approved by the Ethics in Human Research Committees of both study centers.

### Randomization:

Patients were randomized in a 1:1 ratio, with a parallel assignment and unstratified block-randomization approach using an online resource (www.randomizer.org) to receive pyridostigmine or placebo in addition to standard treatment. Placebo and pyridostigmine pill preparation, packing, masking, labeling, and dispensing were performed by trained pharmacy staff at both centers. Participants were enrolled and assigned to treatment by investigators. Throughout the study, participants, site staff, and investigators were masked to the treatment allocation until analysis. There was no formalized evaluation of masking.

### Interventions and outcomes:

The primary outcome was a composite of IMV or death in the 28 days following randomization, and frequency of specific AE. Prespecified secondary outcomes included: the length of hospitalization after randomization; hospital discharge by day-28; the number of days with IMV and the survival rate afterward; failure to complete treatment as expected due to AE; and 90-day mortality.

Participants received either oral pyridostigmine 60 mg/day or a matching placebo for a maximum duration of 14 days, up to hospital discharge, or until the patient had the primary outcome, whichever occurred first, as established *a priori* (Fragoso-Saavedra et al. 2020). We collected demographic information from participants at baseline, including age, sex, and comorbidities. Participants already discharged from the hospital by day 28, including those who were transferred to another facility, were contacted by telephone to assess their status.

AEs were collected using data obtained from the medical records. The events were evaluated and coded using the Medical Dictionary and Regulatory Activities (MedDRA) version 24.0 browser, and the severity of the AEs was assessed by the National Cancer Institute Common Terminology Criteria for Adverse Events, version 5.0.

Although not used throughout the study, unblinding was allowed in case of severe AE at the request of either the physician-treating group or the external Data and Safety Monitoring Board (DSMB). The study was conducted in two parts: phase 2 aimed at determining safety (May 5 to July 4, 2020), followed by phase 3 aimed at evaluating the effect of pyridostigmine in patients with severe COVID-19 (July 5, 2020, to January 30, 2021). Participants in both phases were included in the final study analysis.

### Markers of severe COVID-19

We obtained peripheral venous blood samples on days 0, 3, 5, 7, and 14 after randomization. Samples were collected by certified phlebotomists, processed in the study centers laboratories, and results of complete cell blood count and ferritin, lactate dehydrogenase (LDH), C-reactive protein (CRP), and D-dimer levels were reported to a centralized system and retrieved by the investigators. Plasma was separated by centrifugation and stored immediately at -80 °C. Plasma IL-6, IFN-γ, TNF, and CXCL10 (IP-10) concentrations were measured by sandwich enzyme-linked immunosorbent assay (ELISA). Cytokine concentrations were measured using ELISA MAX deluxe kits (BioLegend, San Diego, CA) following the manufacturer’s instructions. Data were analyzed using the standard curve provided in the kit. Although not a laboratory marker, we also evaluated changes in the qSOFA score to compare the clinical status of the participants throughout the study. The change in values was analyzed considering the baseline and the last recorded measurements, corresponding to the last day of treatment administration.

### Statistical analysis

We planned the study in March 2020, shortly after the report of the first case of COVID-19 in Mexico. At that time, we relied on limited information about clinical outcomes in these patients. Thus, we calculated a sample size of 436 participants considering an event rate of 25% in the control group, a 10% absolute reduction (40% relative reduction) in the primary outcome as clinically significant, and a power of 80% to detect a difference in the primary outcome using a two-sided significance level of α = 0.05. However, we pre-stipulated that the sample size could be adjusted according to interim analyses. The first participant was recruited on May 5, 2020. On July 4, 2020, the DSMB performed a planned interim analysis after the first 44 participants (22 per group, 10% of the calculated sample) to evaluate safety; a second interim analysis was conducted on December 7, 2020, including 28-day outcomes on 100 participants (50 per group). Following the second DSMB interim analysis, the trial was stopped after the recruitment of 188 participants (94 per group) for the following reasons: (1) due to an observed difference in the primary outcome between groups (still blinded to investigators, but unblinded to the DSMB); and, (2) the difficulty in recruiting new participants owing to a reduction in eligible patients related to waning new cases. Considering an α = 0.05, we recalculated the statistical power of the obtained sample with the observed difference between groups (an absolute reduction of 11.7% and a relative reduction of 50%), which resulted in ~ 85%.

We conducted an intention-to-treat analysis for the primary outcome that included all the patients who underwent randomization. As secondary outcomes, we assessed between-group differences in IMV and death with or without IMV. Patients without outcomes by day 28 were censored on day 29. We estimated the effect magnitude of pyridostigmine on the primary outcome as hazard ratio (HR); a 95% confidence interval (CI) was calculated with Cox proportional hazards modeling. We also used a log-rank test to perform multiple posthoc comparisons between groups: IMV, death with or without IMV, and home discharge. We built Kaplan Meier survival curves to plot the cumulative incidence of the primary outcome up to day 28 post-intervention. Statistical analysis was conducted with Prism GraphPad software, version 9.1.0 (GraphPad Software, San Diego, CA), and R version 4.0.0. The full statistical plan is available in the online-material study protocol. To evaluate the differences between both groups regarding laboratory and cytokine values, we performed a repeated-measures ANOVA.

## Results

### Participants

**Fig. 1:**
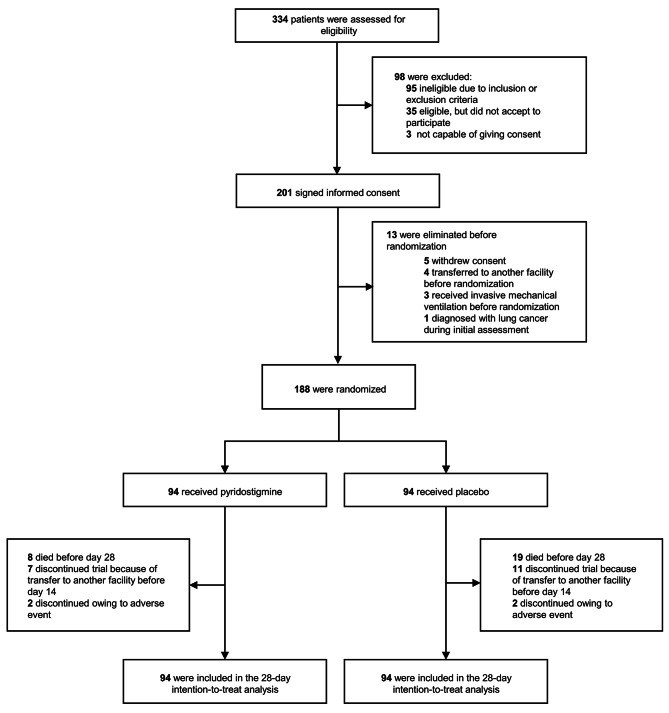
Patient selection flowchart

Between May 5, 2020, and January 30, 2021, we assessed 334 patients for eligibility, of whom 201 accepted to participate; of those, 13 were excluded before randomization for the following reasons: five withdrew consent after signing it; four were transferred to a different facility; three required IMV after giving consent but before randomization, and one was diagnosed with lung cancer (one of our exclusion criteria) after giving consent but before randomization. In total, 188 participants underwent randomization, 94 in each group, and all received at least one dose of the assigned intervention **(**Fig. 1**)**. None of the participants had been previously vaccinated against SARS-CoV-2.

The median age of participants was 52 (interquartile range [IQR], 44–64) years, and 59.6% were men. The most common pre-existing conditions were diabetes and hypertension (Table 1). Patients in the pyridostigmine group had a higher median body mass index (BMI) index (30.1 vs. 28) and lymphocyte counts (856.7 vs. 695.5). All participants had severe COVID-19, were hypoxemic, and received supplementary oxygen as part of in-patient management. The median interval from symptom onset to randomization was 10 (IQR, 9–12) days. The median interval from hospital admission to randomization was 2 (IQR, 1–3) days. Dexamethasone was administered to 148 (78.7%) participants (73 on the pyridostigmine arm and 75 on the placebo arm). Tocilizumab was administered to 11 participants (5 to pyridostigmine recipients and six to placebo recipients). None received remdesivir **(**Table 1 and eTable 1).

**Table 1:** Participant demographics and baseline characteristics

Variable	Placebo	Pyridostigmine	Total
	(*n* = 94)	(*n* = 94)	*(n =* 188)
Sex assigned at birth, *n* (%)			
Male	56 (59.6)	56 (59.6)	112 (59.6)
Female	38 (40.4)	38 (40.4)	76 (40.4)
Age (years), median (IQR)	54 (46–62.5)	51 (43–63)	52 (44–64)
Previous coexisting condition, *n* (%)			
Diabetes	35 (37.2)	33 (35.1)	68 (36.2)
Hypertension	33 (35.1)	33 (35.1)	66 (35.1)
Heart disease	2 (2.1)	2 (2.1)	4 (2.1)
Chronic lung disease	2 (2.1)	6 (6.4)	8 (4.3)
Obesity^a^	31 (33)	50 (53.2)	81 (43.1)
Body mass index (BMI) (kg/m^2^), median (IQR) ^b, c^	28 (25.5–31.3)	30.1 (27.5–33.3)	28.9 (26.1–28.9)
Time (days) from symptom onset to randomization, median (IQR)	10 (9–13)	10 (8–11)	10 (9–12)
Time (days) from admission to randomization, median (IQR)	2 (1–3)	2 (1–2)	2 (1–3)
Oxygen saturation (%), median (IQR)	84 (77–87)	85 (81.5–88)	85 (80–88)
qSOFA score, median (IQR)^d^	1 (0–1)	0 (0–1)	0 (0–1)
qSOFA score = 0, *n* (%)	44 (46.8)	50 (53.1)	94 (50)
qSOFA score = 1, *n* (%)	44 (46.8)	39 (41.4)	83 (44.1)
qSOFA score = 2, *n* (%)	5 (5.3)	5 (5.3)	10 (5.3)
qSOFA score = 3, *n* (%)	0 (0)	0 (0)	0 (0)
Laboratory parameters, median (IQR)			
Lymphocytes, number/µL^a^	695.5 (492.5 -974)	856.7 (580.8 -1323)	773 (519.2–1143)
Neutrophils, number/µL	5932 (4410–9711)	6093 (4085–8813)	5974 (4410–9215)
Monocytes, number/µL	413.5 (281.1–599)	440 (307.8–612.5)	423 (307–599)
D-dimer, µg/mL^e^	672 (423.5–1140)	691.5 (461.8–1005)	679 (460.5–1081)
Ferritin, ng/mL^f^	636.8 (381.1–1046)	482.5 (256.4–906.2)	547.1 (324.5–994.8)
C-reactive protein (CRP), mg/L^g^	12.3 (4.5–19.7)	10.5 (5.5–16.3)	11.4 (5.1–17.8)
Lactate dehydrogenase (LDH), U/L^h^	294 (242–370)	274 (232.3–351)	282 (236–363)
Creatine kinase (CK), IU/L^i^	102 (53–203.5)	94.5 (44–140.3)	96 (51–170)
Proinflammatory cytokines levels (pg/mL), mean (SD)			
IL-6^j^	154 (272.3)	122.7 (206.5)	138 (240.6)
IFN-γ^k^	39.7 (129.3)	24.7 (48.8)	31.8 (96.3)
TNF^l^	2.08 (7.3)	27.3 (114.3)	15.07 (82.8)
CXCL10 (IP-10)^m^	439.1 (261.4)	493.3 (259.6)	469.2 (259.7)
^a^*P ≤ 0.05;*^b^*P ≤ 0.01.*^c^BMI information was not available for 10 patients, four in the pyridostigmine group and six in the placebo group. ^d^Baseline qSOFA score was not available for one patient in the placebo group. ^e^Baseline D-dimer level was not available for one patient in the placebo group and two in the pyridostigmine group. ^f^Baseline ferritin level was not available for two patients in the placebo group and one in the pyridostigmine group. ^g^Baseline CRP level was not available for two patients in the placebo group. ^h^Baseline LDH level was not available for three patients in the placebo group and two in the pyridostigmine group. ^i^Baseline CK level was not available for 13 patients in the placebo group and 12 in the pyridostigmine group. ^j^Baseline IL-6 levels were not available for 23 patients in the placebo group and 20 in the pyridostigmine group. ^k^Baseline IFN-γ level was not available for 24 patients in the placebo group and 20 in the pyridostigmine group. ^l^Baseline TNF level was not available for 23 patients in the placebo group and 19 in the pyridostigmine group. ^m^Baseline CXCL10 (IP-10) level was not available for 66 patients in the placebo group and 59 in the pyridostigmine group.			

One hundred and sixty-six (88.3%) participants received the experimental treatment as planned; 22 (11.7%) discontinued early, nine (9.5%) in the pyridostigmine group, and 13 (13.8%) in the placebo group. Four participants, two in each arm, discontinued trial medication due to mild gastrointestinal symptoms (eTable 2). Eighteen patients discontinued trial medication after being transferred to another medical facility (seven receiving pyridostigmine and 11 receiving placebo) **(**Fig. 1**)**. Precise reasons for transfer were not documented but were likely related to the potential or imminent requirement of critical care; however, in all cases, we were able to determine if an outcome occurred and, if so, the exact date. The median duration of intervention was five (IQR, 3–7) days in the pyridostigmine arm and four (IQR, 3-5.2) in the placebo arm. Adherence was 100% as a physician or nurse supervised each administration and registered it in the medical records.

### Outcomes

**Fig. 2:**
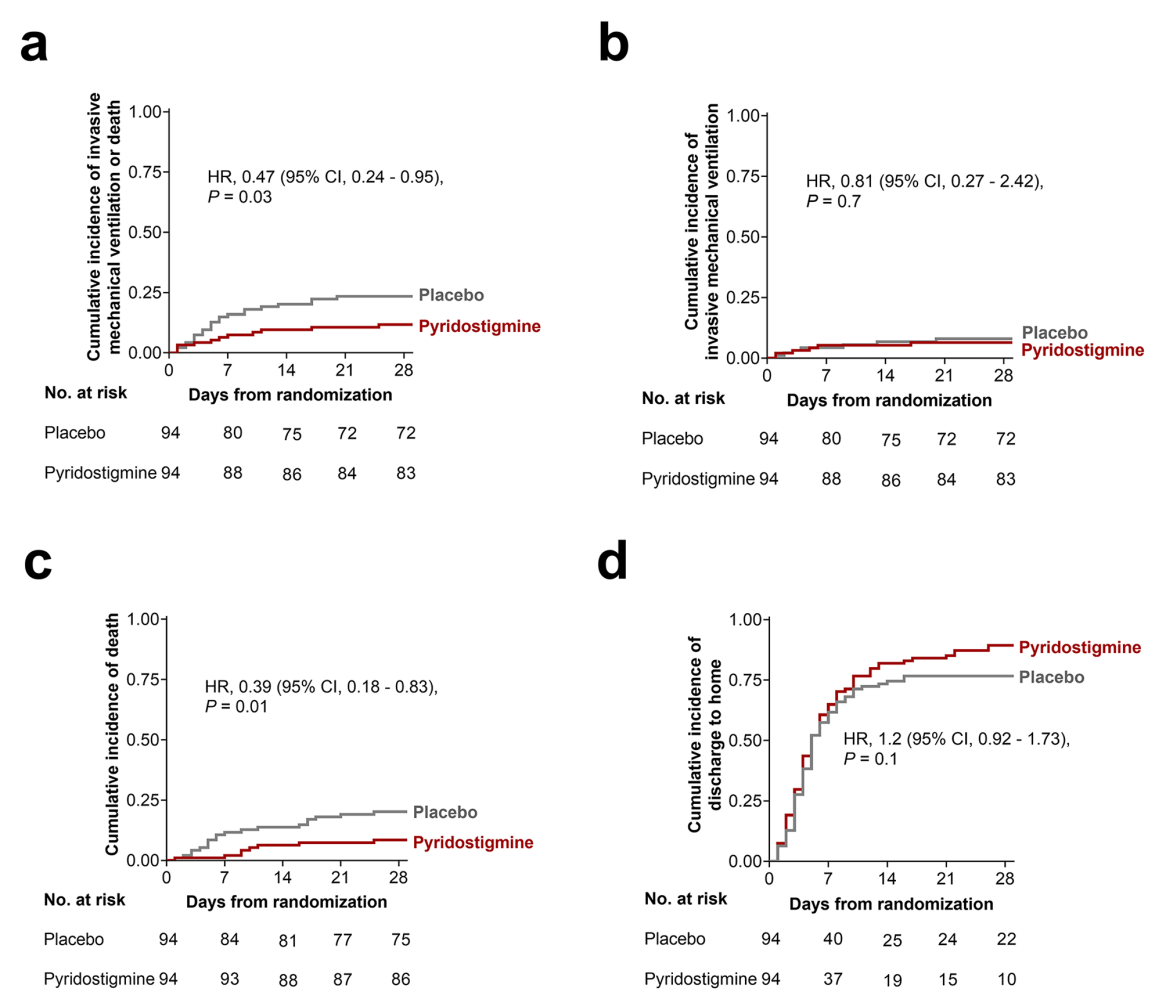
**Pyridostigmine administration reduces mortality in patients with severe COVID-19.** (a) Cumulative incidence of mechanical ventilation or death by day 28 as the composite outcome; (b) cumulative incidence of mechanical ventilation day 28; (c) cumulative incidence of death by day 28;and,(d) cumulative incidence of discharge home by day 28. Hazard ratios were calculated with Cox proportional hazards models and p-values were obtained with a log-rank test. In the case of IMV as the primary outcome, deaths without IMV were censored. In the case of death as the primary outcome, IMV was not considered

Thirty-three participants met the primary outcome by day 28: 11 (11.7%) in the pyridostigmine group, and 22 (23.4%) in the control group (HR 0.47, 95% CI 0.24–0.95, *P* = 0.03) (Fig. 2a**)**. IMV was initiated in 6 (6.3%) pyridostigmine recipients and 7 (7.4%) placebo recipients (HR 0.81, 95%CI 0.27–2.42, *P* = 0.7) (Fig. 2b). The median duration of IMV was 15 (IQR, 15–21) days in the pyridostigmine arm and 17 (IQR, 12–30) days in the placebo arm. The survival rate after IMV was 50% (n = 3) in the pyridostigmine group and 42.9% (n = 3) in the placebo group.

A total of 27 participants died by day 28, eight pyridostigmine recipients and 19 placebo recipients (HR 0.39, 95%CI 0.18–0.83, *P* = 0.01) **(**Fig. 2c**)**. The median in-hospital stay after randomization was 5 (IQR, 3–10) days in the pyridostigmine group and 5 (IQR, 3–8) days in the placebo group (eTable 3). The 28-day hospital discharge rate was 89.3% (84 patients) in the pyridostigmine group and 75.5% (71 patients) in the control group (Fig. 2d). All-cause mortality was also evaluated 90 days after randomization. The only death event after day 28 occurred on day 32 in the pyridostigmine group. Ninety-day mortality was 20.2% (n = 19) in the placebo arm vs. 9.5% (n = 9) in the pyridostigmine arm (HR 0.43, CI 0.2–0.93, *P* = 0.03) (eFigure 11). We identified 77 AEs throughout the study; most were grades 1 and 2. Only six AEs (three in each group) were serious; none was related to the study medication, according to the DSMB. Overall, 42 patients (44.6%) in the pyridostigmine group and 35 patients (37.2%) in the placebo group had at least one AE. The rate and severity of AEs were similar between both groups (eTable 2).

**Fig. 3:**
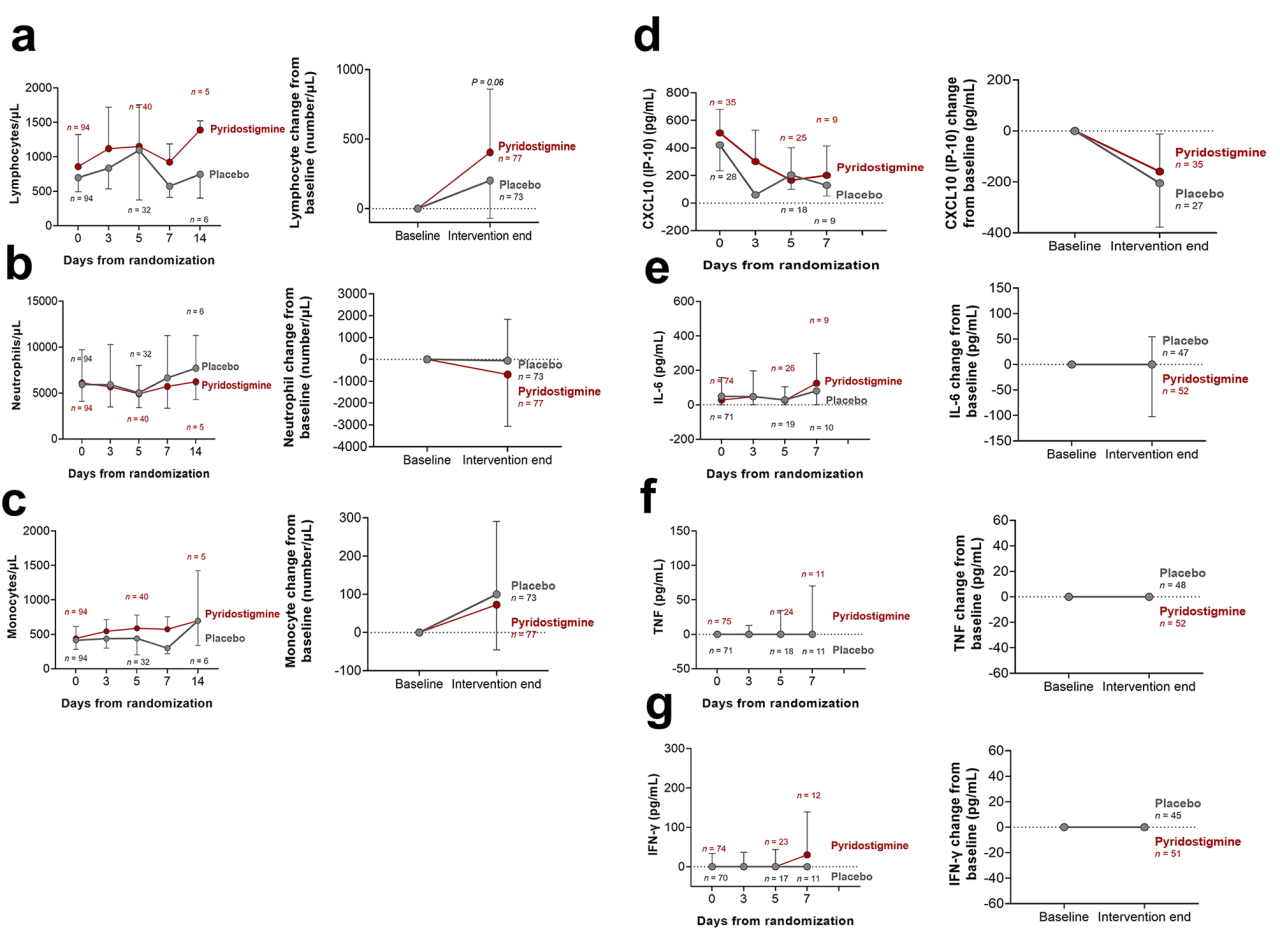
**Between-group time course of circulating hematological and inflammatory markers of severity.** Circulating lymphocyte (a), neutrophil (b), monocyte (c), CXCL-10 (d), IL-6 (e), TNF (f), and IFN-γ (g) values. Sample sizes at each time point are indicated. All data are shown as median ± IQR. Only the upper quartile for the group with the highest value and the lower quartile for the group with the smallest value are shown to avoid image cluttering

A difference in baseline laboratory values was observed regarding lymphocyte counts, TNF, and ferritin (Table 1). However, those differences were not considered clinically relevant. Regarding repeated measures of laboratory values, we did not observe a clinically relevant effect of the treatment arm regarding changes in lymphocyte, neutrophil, or monocyte counts, as well as in circulating levels of CXCL-10, IL-6, TNF, IFN-γ, d-dimer, ferritin, CRP, or LDH, as measured by eta^2^ (Fig. 3, eFigure 2, and eTable 4).

As obesity status differed between groups, we performed a sensitivity analysis with a Cox proportional hazards model adjusted for obesity. For the sensitivity analysis, we excluded ten patients (four pyridostigmine recipients and six placebo recipients) that had no records of their weight. Among the excluded patients, only one reached the primary outcome. We obtained an adjusted HR of 0.42 (95% CI 0.19–0.91, *P* = 0.05) for the primary outcome; 0.78 (95%CI 0.24–2.54, *P* = 0.3) for IMV; and 0.32 (95%CI 0.13–0.77, *P* = 0.03) for death. Outcomes were uniformly distributed across the study period. However, given dexamethasone was not routinely used during the first part of the trial, we performed an additional sensitivity analysis to determine if dexamethasone could modify the effect of pyridostigmine. A Cox regression model with “dexamethasone” as a covariate was built. The resulting HR for the primary outcome was 0.45 (95% CI 0.22–0.94) favoring pyridostigmine.

## Discussion

Recent studies show that the in-hospital mortality rate in COVID-19 still ranges from 20 to over 40% despite the overall decline throughout the pandemic (Rossman et al. 2021; Olivas-Martínez et al. 2021; Souza et al. 2021; Núñez et al. 2021). The severity and mortality of COVID-19 are mediated by an uncontrolled inflammatory response to infection (Mehta et al. 2020). Hence, along with the finding of immunomodulatory interventions, the repurposing of drugs with well-characterized safety profiles and readily available production lines such as pyridostigmine might lead to faster development of anti-COVID-19 therapies. Here, we show that added to standard medical care, pyridostigmine is associated with reduced mortality in patients hospitalized for severe COVID-19.

In this trial, we observed a reduction in the primary outcome of IMV or death in 11.7% of pyridostigmine recipients. When analyzed separately, we saw that this effect was driven by a reduction in mortality (19 vs. 8 deaths, respectively) with no differences in IMV incidence. This incidence of death is lower than the one reported in the active arms of other therapeutic trials. For instance, the risk of death still exceeded 20% in the dexamethasone and tocilizumab arms of the RECOVERY trial(Horby et al. 2021a, b). This may be explained by many factors, including genetic and environmental factors, or improved care as a product of cumulative experience in treating severe COVID-19. Similarly, in the placebo group of our study, we observed 28-day mortality of 20.2%, but a majority (78.7%) received dexamethasone as part of the standard of care, indicating that, without further intervention, in-hospital mortality of severe COVID-19 remains high.

As the inflammatory response influences the clinical outcomes in severe COVID-19, we evaluated blood markers associated with a higher probability of respiratory failure and death (Núñez et al. 2021; Wang et al. 2021; Ouyang et al. 2020). In this trial, pyridostigmine did not lead to a relevant recovery of neutrophilia or rescue from lymphopenia. Also, pyridostigmine did not reduce the circulating concentrations of acute-phase reactants and pro-inflammatory cytokines. Previous studies in people living with human immunodeficiency virus showed pyridostigmine did reduce IFN-γ, but not other cytokines(Valdés-Ferrer et al. 2009). Thus, the lack of inflammatory marker reduction we observed does not directly contradict the results of previous studies. Experiments in murine ARDS models demonstrate the utility of pyridostigmine and α7-nACh-R agonists to reduce lung and systemic inflammation as well as mortality (Su et al. 2010; Pinheiro et al. 2021; Bricher Choque et al. 2021). The reduction in mortality we observed in patients who received dexamethasone plus pyridostigmine, may suggest a synergistic immunomodulatory effect; however, the study was not designed -and is underpowered- to answer that question.

The strengths of our study include that it is a double-blinded, placebo-controlled, multicentric randomized controlled trial, which evaluated an inexpensive and safe therapeutic intervention, with a positive pharmacological profile, and wide availability as a generic drug. The demographics of our study population, including age and comorbidities, are representative of a large group of the underserved population worldwide, which is in urgent need of safe, life-saving, and affordable treatments for severe COVID-19. Moreover, with the periodic emergence of novel variants, new outbreaks keep appearing, including breakthrough cases among vaccinated individuals. Hence, novel, accessible, and safe treatments are urgently needed.

Our study has some limitations. The study was halted early by the recommendation of the DSMB due to a greater-than-expected effect of the treatment arm and due to difficulties in recruitment related to a decrease in severe COVID-19 cases. Also, some patients requiring IMV could probably not receive it because of a pre-existing living will, last-minute patient (or proxy) refusal of intubation, or, intermittently throughout the study, lack of availability of critical care beds (Olivas-Martínez et al. 2021). Resources varied daily, and sometimes even considerably at different times during the same day. However, if we analyze each portion of the outcome separately, we observe that among 19 deaths in the placebo group, 15 occurred without receiving mechanical ventilation (~ 79%), while 5 of 8 patients in the pyridostigmine group died without receiving mechanical ventilation (~ 63%). Bed availability and who got to be admitted were not influenced in any way by the study investigators, so any variation in this regard is purely by chance. Our primary outcome included initiation, but not necessarily the requirement of IMV. This is reflected by the fact that the bulk of the primary outcome was due to deaths among participants who did not receive IMV, most likely related to a lack of critical care space. Thus, our results may not be generalizable to settings that do not have this problem; however, healthcare strain continues to occur worldwide as new surges in cases occur, particularly among those who refuse vaccines, which makes our results potentially generalizable (Núñez et al. 2022). The recruitment of participants for our study lasted eight months. When we designed the study, no effective interventions existed. The most relevant change in COVID-19 treatment during that period was the inclusion of dexamethasone. Thus, the sub-analysis performed among people who received dexamethasone was unplanned, limiting the conclusions that can be drawn from it. While most baseline characteristics were evenly balanced between groups, obesity (a known predictor of adverse outcomes in COVID-19) was randomly more prevalent in the pyridostigmine group; this imbalance that in principle plays against pyridostigmine strengthens our observed outcomes. As a proof-of-concept one, this study needs to be replicated or refuted in independent RCTs. Also, our results cannot be extrapolated to patients with less severe COVID-19, or those already receiving IMV, without prior verification in RCTs.

## Conclusion

Here, we present evidence to support the use of pyridostigmine as an add-on treatment to standard care in patients hospitalized for severe COVID-19. Our data indicate that such an inexpensive and readily available drug may significantly reduce mortality without imposing relevant adverse events.

## Data Availability

The datasets analyzed during the current study are available from the corresponding author upon reasonable request.

## Declarations

### Trial registration

ClinicalTrials.gov, identifier: NCT04343963.

## Abbreviations

COVID-19: Coronavirus disease 2019.
Ach: Acetylcholine.
IMV: Invasive mechanical ventilation.
AE: Adverse events.
IQR: Interquartile range.
SARS-CoV-2: Severe acute respiratory syndrome coronavirus 2.
ARDS: Acute respiratory distress syndrome.
α7-nACh-R: α-7 nicotinic ACh receptors.
ChAT: Choline-acetyltransferase.
i-ACh-e: Acetylcholinesterase inhibitor.
HIV: Human immunodeficiency virus.
RT-PCR: Real-time polymerase-chain-reaction.
DSM: Data and Safety Monitoring Board.
LDH: lactate dehydrogenase.
CRP: C-reactive protein.
ELISA: Enzyme-linked immunosorbent assay.
BMI: Body mass index.
HR: Hazard ratio.
RCT: Randomized controlled trial.
